# Bacteriophage EPP-1, a potential antibiotic alternative for controlling edwardsiellosis caused by *Edwardsiella piscicida* while mitigating drug-resistant gene dissemination

**DOI:** 10.1038/s41598-024-60214-3

**Published:** 2024-04-24

**Authors:** Ganghua Han, Ting Huang, Xinchun Liu, Ruyin Liu

**Affiliations:** 1 College of Resources and Environment, University of Chinese Academy of Sciences, No. 19(A) Yuquan Road, Shijingshan District, Beijing, 100049 People’s Republic of China; 2 Yanshan Earth Critical Zone National Research Station, University of Chinese Academy of Sciences, Beijing, People’s Republic of China

**Keywords:** Phage therapy, Drug-resistant gene, *Edwardsiella piscicida*, Edwardsiellosis, Antibiotic alternative, Biotechnology, Microbiology, Ecology, Environmental sciences

## Abstract

*Edwardsiella piscicida* causes significant economic losses to the aquaculture industry worldwide. Phage-based biocontrol methods are experiencing a renaissance because of the spread of drug-resistant genes and bacteria resulting from the heavy use of antibiotics. Here, we showed that the novel *Edwardsiella* phage EPP-1 could achieve comparable efficacy to florfenicol using a zebrafish model of *Edwardsiella piscicida* infection and could reduce the content of the *floR* resistance gene in zebrafish excreta. Specifically, phage EPP-1 inhibited bacterial growth in vitro and significantly improved the zebrafish survival rate in vivo (*P* = 0.0035), achieving an efficacy comparable to that of florfenicol (*P* = 0.2304). Notably, integrating the results of 16S rRNA sequencing, metagenomic sequencing, and qPCR, although the effects of phage EPP-1 converged with those of florfenicol in terms of the community composition and potential function of the zebrafish gut microbiota, it reduced the *floR* gene content in zebrafish excreta and aquaculture water. Overall, our study highlights the feasibility and safety of phage therapy for edwardsiellosis control, which has profound implications for the development of antibiotic alternatives to address the antibiotic crisis.

## Introduction

*Edwardsiella piscicida* is a notorious fish pathogen that infects its host mainly through the gut^1^, leading to severe edwardsiellosis and causing substantial economic losses to the aquaculture industry worldwide^2,3^. The Food and Drug Administration (FDA) recommends a dose of 10‒15 mg florfenicol/kg fish for no more than 10 consecutive days to combat edwardsiellosis in aquaculture (https:// www.fda.gov/media/84229/download). Recent studies, however, have unveiled that exposure to standard therapeutic doses of florfenicol alters the structure of the gut microbiota and increase the abundance of florfenicol-resistance genes in aquaculture systems^4,5^. The use of other antibiotics like oxytetracycline, sulfamethoxazole, or enrofloxacin similarly induced rise of antibiotic resistance gene (ARG) levels or emergence of new antibiotic resistant bacteria (ARB) across a wide range of habitats^6,7^. These ARGs or ARB possess the potential to disseminate into the environment^8–10^, thereby heightening their risks in global public health sector^11,12^. Accordingly, the excessive use of antibiotics overshadows the sustainable development of the aquaculture industry, and particularly in the global context of “One Health,” novel antibacterial agents are urgently needed.

Bacteriophages (phages), viruses that specifically infect bacteria, are ubiquitous and abundant worldwide, with an estimated total number exceeding 10^31^ particles^13^. Phage-based biocontrol methods, or phage therapy, have been reinvigorated with unprecedented momentum and are emerging as important strategies in the post-antibiotic era due to the fact that it can reduce the use of antibiotics at the source and alleviate a series of issues caused by the use of antibiotics^14,15^. Phage therapy in the field of aquaculture is now being researched against *Vibrio*, *Flavobacterium*, *Aeromonas*, *Pseudomonas*, and *Lactococcus* and has yielded favorable outcomes^16,17^. Available evidence suggests that administration of *E. tarda* phage vB_EtaM_ET-ABTNL-9 by feeding, injection, or immersion significantly reduced mortality as well as nonspecific immune-related enzyme activities of turbot *Scophthalmus maximus*^18^. Furthermore, a phage cocktail composed of *E. tarda* phage PETp9 and *V. harveyi* phage PVHp5 significantly reduced host levels of pathogens and maintained the normal gut microbiota profile for ascites prevention in turbot^19^. However, a comprehensive evaluation of the therapeutic efficacy and safety of phages for the biocontrol of edwardsiellosis caused by *E. piscicida* infections is still lacking. More importantly, as an antibiotic alternative, the reduction extent of resistance genes by phages during treatment has also not been thoroughly assessed. Elucidation of these issues is a prerequisite for implementing phage therapy in aquaculture and addressing the associated antibiotic resistance issues.

Zebrafish (*Danio rerio*) is the most commonly used model species for environmental monitoring and toxicological evaluations^20,21^. Therefore, it was selected as the experimental subject in this study. Here, we isolated a novel *E. piscicida* phage and characterized its physiological and genomic properties. We also investigated its bactericidal effect on *E. piscicida *in vitro and its protective effect on zebrafish in vivo. In addition, we evaluated the safety of phage therapy for edwardsiellosis control in zebrafish in terms of the antioxidant capacity and inflammatory cytokines in the gut and liver of the model species, the diversity and function of the gut microbial community, and the content of the *floR* gene in fish excreta and environmental media. To the best of our knowledge, this is the first attempt to use a novel phage to control edwardsiellosis caused by *E. piscicida* and to comprehensively evaluate its safety for aquaculture. This study thus provides novel insights into the biocontrol of edwardsiellosis in aquaculture and has profound implications for the development of antibiotic alternatives to address the antibiotic crisis.

## Materials and methods

### Bacterial strains and zebrafish culture

Bacteria used in this study (details are provided in Table S1) were obtained from the Marine Culture Collection of China (MCCC) and cultivated in Trypticase Soy Broth medium (Solarbio, Beijing, China) at 30℃ with 120 rpm of shaking. Our PCR results showed that the *floR* gene was not detected in any of the strains^22^. In addition, the antimicrobial susceptibility test showed that even at concentrations as low as 5 μg/mL, florfenicol can effectively inhibit the growth of host *E. piscicida* used in this study (Fig. S1). Wild-type zebrafish (*D. rerio*, AB line, 3‒4 cm, 0.3‒0.4 g) were purchased from Zhongke Water Quality Environmental Technology Co., Ltd. (Wuxi, Jiangsu, China), raised in a standard aquaculture system with a photoperiod of 14 h:10 h light/dark, and fed *Artemia salina* twice daily. To avoid water quality deterioration and potential impacts on subsequent experiments, one-third of the aquaculture water was replaced daily with tap water that was aerated for 24 h, water quality (Table S2) was monitored every 2 days, and feces produced by zebrafish was collected thoroughly on days 2, 4, and 7 during the whole experiment. All zebrafish used in this study were acclimatized for at least seven days under the standard aquaculture system prior to the next step in the experiment, and the conditions were consistent throughout the experiment.

### Phage isolation, characterization, and genome analysis

The double-layer agar method, as described by Thung^23^, was used to isolate *Edwardsiella* phages from aquaculture wastewater and natural surface water (Supplementary Information). After purification and proliferation, several characteristics of the isolated phages were characterized, including morphology via transmission electron microscopy (TEM), host spectrum, and temperature and pH tolerance (see Supplementary Information for details). Phage nucleic acids were extracted and purified, as described by Kim et al^24^. (Supplementary Information). Purified phage DNA was randomly broken into 350 bp fragments and then linked to a specific adapter for library preparation using the standard NEBNext® Ultra™ II DNA Library Preparation Kit for Illumina®. After checking the library, paired-end 2 × 150 sequencing was performed on an Illumina NovaSeq platform (Illumina, San Diego, CA, USA) at Fixgene Technology Co., Ltd. (Beijing, China). The raw sequencing data were subjected to quality control using fastp^25^ and then assembled using SPAdes (v.3.12.0)^26^. The resulting sequences were corrected using PhageTerm^27^. Subsequent bioinformatic analysis is presented in the Supplementary Information.

### Phage therapy versus antibiotic therapy for edwardsiellosis

To compare the efficacy of phage therapy with that of conventional antibiotic therapy for edwardsiellosis control in the zebrafish model, we set up four different treatment scenarios based on intraperitoneal injection, namely the PBS + SM group (negative control group, PBS buffer (137 mM of NaCl, 2.7 mM of KCl, 10 mM of Na_2_HPO_4_, 1.76 mM of KH_2_PO_4_, pH = 7.4) + SM buffer (200 mM of NaCl, 10 mM of MgSO_4_, 50 mM of Tris–HCl, pH = 7.5)), *E. p.* + SM group (positive control group, 10^5^ CFU *E. piscicida* (MCCC 1K00246)/fish, determined in the study), *E. p.* + FLO group (florfenicol therapy group, 10^5^ CFU *E. piscicida* (MCCC 1K00246)/fish + 10 mg florfenicol/kg fish weight), and *E. p.* + EPP-1 group (phage therapy group, 10^5^ CFU *E. piscicida* (MCCC 1K00246)/fish + MOI of 1 for the phage EPP-1, determined in the study), with 30 zebrafish per tank. In the described above, the *E. piscicida* was diluted with PBS, phage EPP-1 and florfenicol were diluted with SM buffer. The number of dead zebrafish was recorded daily for seven consecutive days to plot survival curves. The effects of the different treatments on the antioxidant capacity and levels of inflammatory cytokines in the gut and liver of zebrafish were evaluated. Finally, zebrafish feces and farmed water were collected on days 2, 4, and 7 for microbial-related analyses.

#### Oxidative stress and inflammatory cytokines in the gut and liver

On days 1, 3, and 7 after intraperitoneal injection, 6 zebrafish were randomly selected and divided into two groups with three biological replicates each, one group for the antioxidant capacity assay and the other group for the inflammatory cytokine assay. Briefly, the zebrafish were sacrificed to harvest the gut and liver samples. These samples were then weighed and prepared as 10% homogenates with saline (for antioxidant capacity determination) and PBS (for inflammatory cytokine determination) and centrifuged at 6000 × *g* for 10 min, and the supernatant was collected. Superoxide dismutase (SOD) and catalase (CAT) activity and reduced glutathione (GSH) content were determined using SOD, CAT, and GSH assay kits (Nanjing Jiancheng Bioeng Inst., Nanjing, China). Inflammatory cytokines, including interleukin-1β (IL-1β), interleukin-6 (IL-6), tumor necrosis factor-α (TNF-α), and interferon-γ (IFN-γ) were quantified using fish tissue-specific enzyme-linked immunosorbent assay kits (Nanjing Jiancheng Bioeng Inst., Nanjing, China). Enzyme activity and inflammatory cytokine levels were determined according to the manufacturer’s instructions using a microplate reader (Synergy™ H1; BioTek Inc., Winooski, VT, USA) at specific wavelengths. Each measurement was conducted in triple biological replication. T-test analysis was performed to examine the difference among different treatments.

#### Nucleic acid extraction from water and zebrafish feces

Feed water and zebrafish fecal samples were collected from different treatment groups for DNA extraction on days 2, 4, and 7 after intraperitoneal injection, that is, 3 water samples and 3 fecal samples for each treatment. Approximately 600 mL of water samples were filtered through 0.22 μm-pore-size membranes (Merck Millipore, Bedford, MA, USA) within 24 h, and the membranes were then cut with sterile scissors for DNA extraction. Fecal samples were aspirated from the bottom of the tank, centrifuged at 6000 × *g* for 10 min, washed three times with PBS, and weighed, after which they were ready for DNA extraction. Genomic DNA was extracted from water and fecal samples using the FastDNA™ Spin Kit for Soil (MP Biomedicals, Santa Ana, CA, USA), following the manufacturer’s instructions. Finally, the extracted DNA was fractionated and stored at − 20°C for further analysis.

#### Quantification of E. piscicida and the floR gene

The host pathogen *E. piscicida* and the florfenicol-resistance gene *floR* were measured in feeding water and zebrafish feces via quantitative polymerase chain reaction (qPCR) using previously reported available primers (*gyrB* and *floR* genes for *E. piscicida* and florfenicol-resistance gene quantification, respectively) and programs^22^, respectively. A standard curve was first plotted based on constructed plasmids with known content of target genes, and then the content of target genes in the samples was calculated based on this standard curve. The primer sequences, product sizes, and detailed qPCR programs are shown in Supplementary Information Table S3. Three replicates for each are used for quantification and the content of each gene is presented as the mean ± standard deviation.

#### 16S rRNA gene sequencing and analysis

The V3–V4 hypervariable regions of the bacterial 16S rRNA gene were amplified via PCR using the barcoded primer pair 338F and 806R (Table S3). Amplicon sequencing was performed on an Illumina MiSeq platform (Illumina, Woburn, MA, USA) by Shanghai Majorbio Bio-pharm Technology Co., Ltd. (Shanghai, China), and sequencing data were analyzed using the online Majorbio Cloud Platform (https://cloud.majorbio.com/)^28^. Briefly, the raw paired sequences were subjected to quality control and merged using fastp (v0.19.6)^25^ and FLASH (v1.2.11)^29^. The resulting tags were analyzed using QIIME 1.9.1^30^, following the steps reported by Guo et al^31^. The quality-filtered sequences were clustered into operational taxonomic units (OTUs) with a 97% identity cut-off using UPARSE (v11)^32^ and annotated based on taxonomy with the Silva (SSU138) 16S rRNA database as a reference, with a 70% classification confidence threshold^33^, using the naïve Bayesian-based RDP Classifier (v2.13)^34^. Alpha diversity indices and Bray–Curtis distance-based beta diversity indices were calculated using QIIME 1.9.1^30^. Principal coordinate analysis (PCoA) and non-metric multidimensional scaling (NMDS) analysis at the OTU level were performed using RStudio according to the Bray–Curtis distance matrix.

#### Metagenomic analysis of the gut microbial communities

Metagenomic sequencing of 12 fecal samples was conducted on an Illumina HiSeq 4000 platform (Illumina, Woburn, MA, USA) with a 2 × 150 bp paired-end sequencing strategy by Shanghai Majorbio Bio-pharm Technology Co., Ltd. (Shanghai, China). Adapters, sequences with a length < 50 bp or an average quality score < 20, or reads containing the “N” base were removed from raw reads using fastp (v0.20.0)^25^. The resulting clean reads were assembled using the succinct de Bruijn graph method in Megahit (v1.1.2)^35^. Open reading frames (ORFs) were predicted from assembled contigs > 100 bp using Prodigal (v2.6.3)^36^. The predicted ORFs were clustered using CD-HIT (v4.6.1)^37^ with 90% identity and 90% coverage, to construct a non-redundant gene set. The high-quality reads of each sample were then matched to the gene set using SOAPaligner (v2.21)^38^, based on the Reads Per Kilobase per Million (RPKM) algorithm^39^, with 95% identity to determine the abundance of target genes. Gene function was annotated by aligning the sequences of the non-redundant gene set against the Kyoto Encyclopedia of Genes and Genomes (KEGG) database using DIAMOND (v0.8.35)^40^ with an E-value ≤ 10^−5^. Carbohydrate-active enzymes and antibiotic resistance genes were annotated, and their abundances were calculated based on the Carbohydrate-Active enZYmes Database (CAZy)^41^ and the Comprehensive Antibiotic Resistance Database (CARD)^42^, respectively. In addition, PCoA, NMDS, and Kruskal–Wallis H tests of gene function at multiple taxonomic levels were performed based on RPKM abundance using the Bray–Curtis distance matrix.

### Statistical analysis

Student’s *t*-test, One-way ANOVA with Sidak’s post-hoc test, and two-way ANOVA were performed in IBM SPSS (v25.0). Survival analyses with Mantel-Cox test was performed in Graphpad Prism (v9.0). *P* < 0.05 was considered statistically significant. Stress value < 0.05 in NMDS indicates their good conformity.

### Data availability

All sequencing data have been deposited in the NCBI Sequence Read Archive under the BioProject ID PRJNA964478.

### Ethics in publishing

All zebrafish experiments in this work were in accordance with the National Research Council's Guide for the Care and Use of Laboratory Animals. This work has received approval for research ethics from the Institutional Animal Care at University of Chinese Academy of Sciences, where the experiment was conducted. All experiments were performed in accordance with ARRIVE guidelines (https://arriveguidelines.org).

## Results

### Isolation and characterization of phage EPP-1

**Figure 1:**
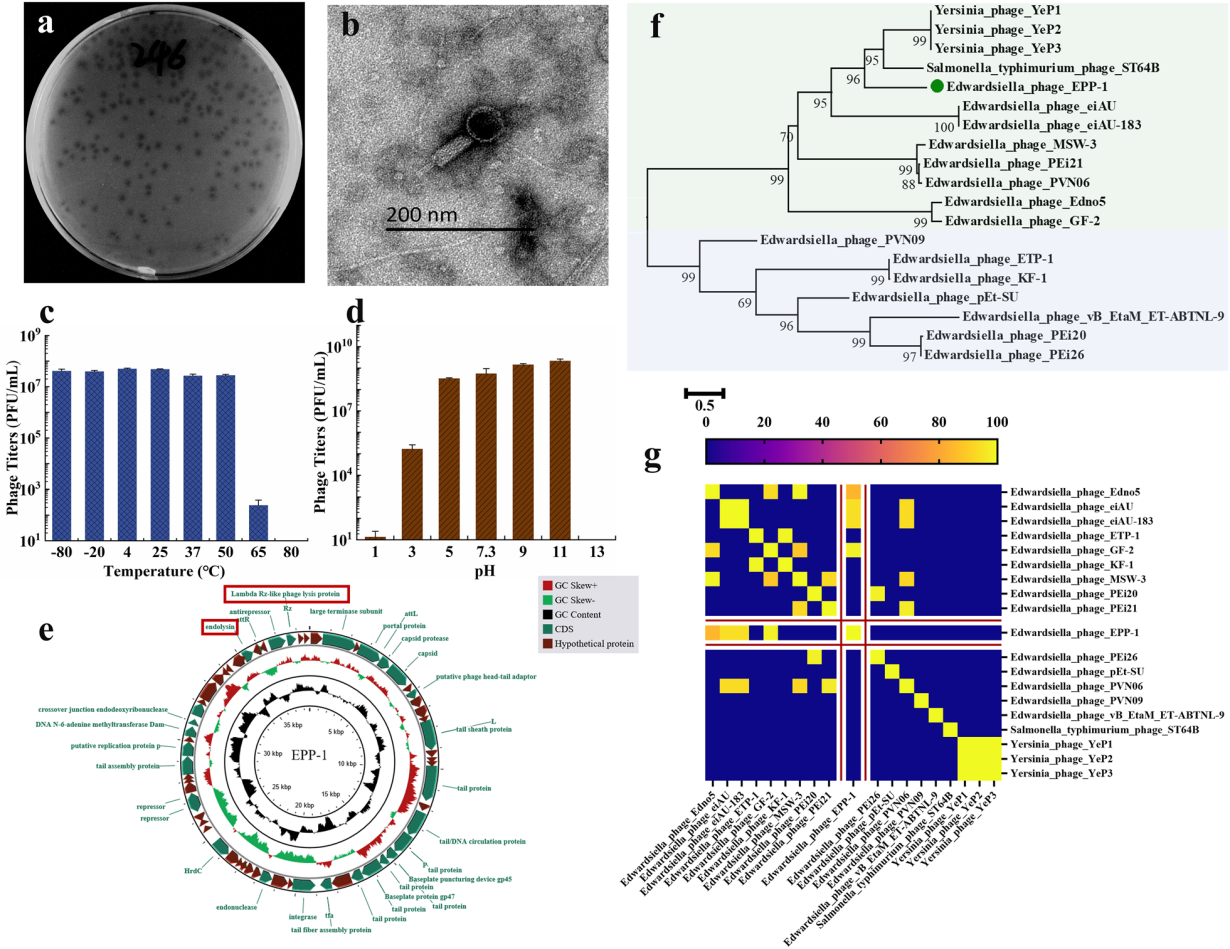
The morphology, physiology, phylogenetic and genomic characteristics of phage EPP-1. (**a**) Clear plaques formed by phage EPP-1 on the double layer plate, and (**b**) the morphology of phage EPP-1 under TEM. (**c**) Temperature and (**d**) pH tolerance of phage EPP-1, shown as mean ± SD (n = 3). (**e**) The circle genetic map of phage EPP-1. GC skew and GC content are shown in the inner circle, and the CDSs predicted are shown in the outer circle. The green pattern represents CDSs of known function and the brown pattern represents hypothetical proteins. (**f**) Phylogenetic tree of phage EPP-1 based on the *TerL* gene. (**g**) Heatmap of the ANI values calculated using 19 related phage genomes.

In this study, an *Edwardsiella* phage, named EPP-1, was isolated from aquaculture wastewater in Henan, China, and preserved at the China General Microbiological Culture Collection Center (CGMCC; preservation number, CGMCC No. 45078). The complete genome of the phage was uploaded to NCBI with GenBank accession number OQ910326. Phage EPP-1 lysed host *E. piscicida* MCCC 1K00246 and formed clear plaques on a double-layer plate (Fig. 1a). TEM-based morphology showed that phage EPP-1 has a head of 37 nm in diameter and a tail that is 103 nm in length (Fig. 1b). The host range of phage EPP-1 identified by spot assay showed that this phage also lysed *E. piscicida* MCCC 1K03230, *E. tarda* MCCC 1K00241, *Edwardsiella* sp. MCCC 1K00239, *Edwardsiella* sp. MCCC 1K00240, and *Edwardsiella* sp. MCCC 1K00242, indicating their broad-spectrum properties (Table S1). Phage EPP-1 was found to have strong tolerance to low and moderate temperatures; its titer began to decrease at temperatures above 50℃ and completely vanished at 80℃ (Fig. 1c). The phage was highly stable between pH 5 and 11, but its numbers decreased from 8.51 log_10_ PFU/mL at pH 5 to 3.48 log_10_ PFU/mL at pH 3, and it was completely inactivated under more acidic or alkaline conditions (Fig. 1d).

According to whole-genome sequencing, the genome of phage EPP-1 was structured as linear dsDNA, with a size of 38,641 bp and a GC content of 52.29%. PHASTER predicted 66 CDSs from the EPP-1 genome, of which 28 appeared to encode functional proteins, such as phage structural proteins, DNA replication-related enzymes, and lysis-related enzymes. Notably, lysogeny-related genes, such as the integrase and recombination loci *attL* and *attR*, were also predicted in the phage EPP-1 genome (Fig. 1e), suggesting that the virus might enter the lysogenic cycle in phage–host interactions. No virulence factors, ARGs, or tRNA were identified in the genome of EPP-1. According to *TerL*-based phylogenic analysis, phage EPP-1 was distantly related to other *Edwardsiella* phages but closely related to the *Yersinia* phages YeP1, YeP2, and YeP3 and *Salmonella typhimurium* phage ST64B (Fig. 1f). The average nucleotide identity (ANI) values for whole-genome comparisons were calculated using the same genome collections. The highest ANI value was 98.11% for phage EPP-1 with *Edwardsiella* phage GF-2, which has a genome size of 43,129 bp, whereas the remaining ANI values were lower than 95% (Fig. 1g), indicating the distinctiveness of phage EPP-1 isolated in this study.

### Phage EPP-1 treatment is comparable to florfenicol in efficacy

**Figure 2:**
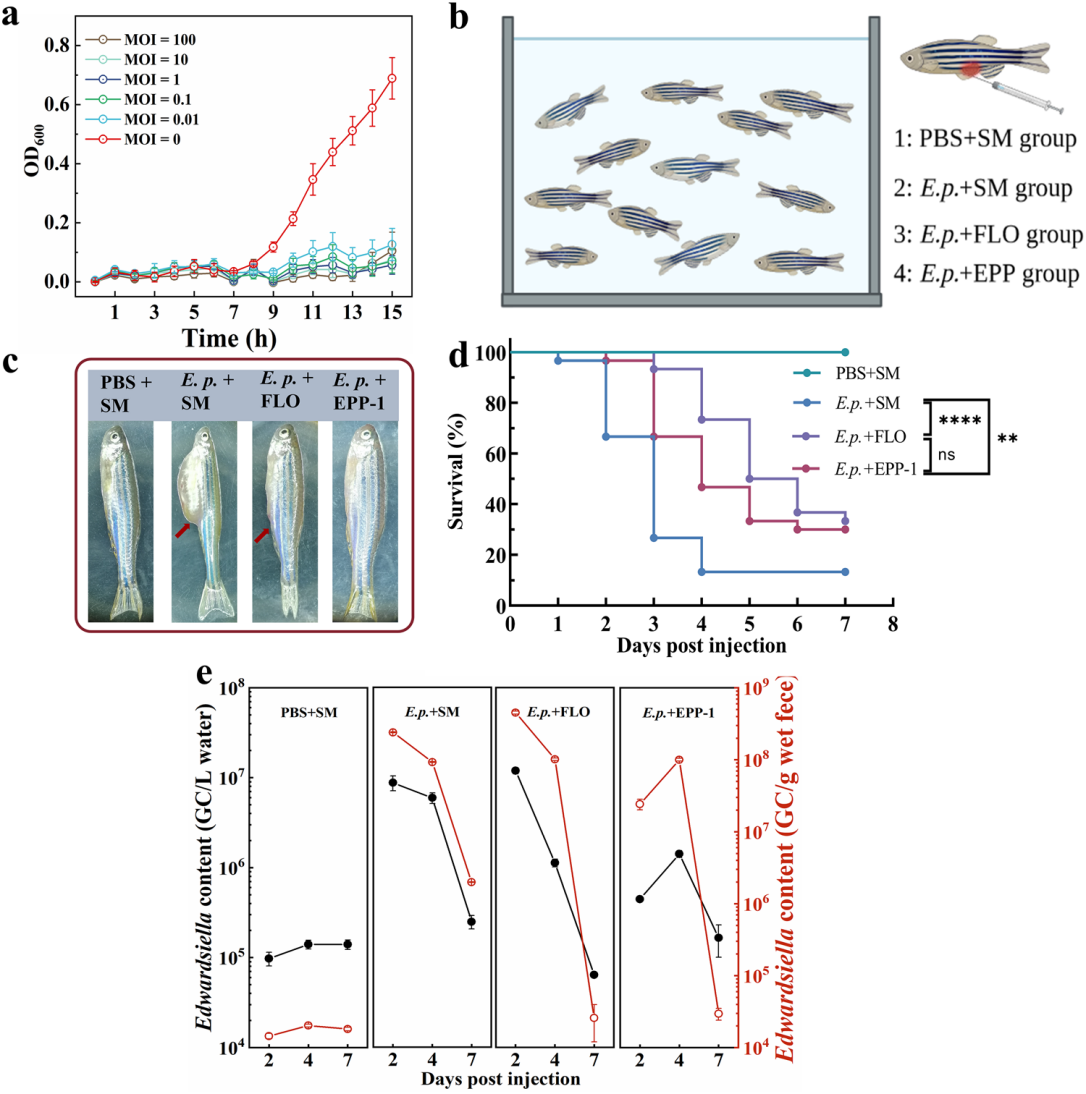
Comparison of the efficacy of phage EPP-1 and florfenicol treatments. (**a**) The bactericidal effects of phage EPP-1 under different MOIs in vitro. (**b**) Experimental setup for in vivo comparison of phage EPP-1 and florfenicol treatments. (**c**) Clinical symptoms of zebrafish under different treatments on day 3. (**d**) Survival curves of zebrafish under different treatments within 7 days (n = 30, *P < 0.05, **P < 0.01, ***P < 0.001, ****P < 0.0001, Mantel-Cox test). (**e**) The qPCR results of *Edwardsiella* content in water and zebrafish feces.

The in vitro antibacterial effect of phage EPP-1 and its protective effect in vivo in a zebrafish model were investigated to evaluate its feasibility for use in edwardsiellosis control in aquaculture. Phage EPP-1 showed strong antibacterial activity in vitro, effectively inhibiting the growth of its host, making their OD_600_ values lower than 0.2 even at a low MOI of 0.01, as a comparison, the group without phage addition had an OD_600_ value of more than 0.6 (Fig. 2a). For in vivo assays, 10^5^ CFU/fish of *E. piscicida* 1K00246 was used (Fig. S2a). Given the complex components of the zebrafish gut, which contribute to a harsher condition than that used in the in vitro assay, different doses (MOIs of 0, 0.1, 1, 5, and 10) of phage EPP-1 were administered to determine the optimal therapeutic dose. An MOI of 1 significantly improved the zebrafish survival compared to that in the challenge group (MOI = 1 *vs* challenge group, *P* = 0.0008) and was comparable to that in the higher dose groups (MOI = 1 *vs* MOI = 5, *P* = 0.6491; MOI = 1 *vs* MOI = 10, *P* = 0.1572) (Fig. S2b), and thus was selected for further trials.

To compare the therapeutic efficacy of phage EPP-1 with that of the antibiotic florfenicol for edwardsiellosis control, four different treatment scenarios were established as follows: PBS + SM negative control group, *E. p.* + SM positive control group, *E. p.* + FLO treatment group, and *E. p.* + EPP-1 treatment group (Fig. 2b). Phage EPP-1 alleviated the signs of ascites in zebrafish caused by *E. piscicida* infection (Fig. 2c). Both treatment groups showed significantly decreased zebrafish mortality (*E. p.* + SM *vs E. p.* + EPP-1, *P* = 0.0035; *E. p.* + SM *vs E. p.* + FLO, *P* < 0.0001), and the efficacy of phage EPP-1 was comparable to that of conventional florfenicol (*E. p.* + FLO *vs E. p.* + EPP-1, *P* = 0.2304) (Fig. 2d).

Pathogenic *E. piscicida*, introduced into the guts of zebrafish, can enter the environment via excrement. *Edwardsiella*-specific qPCR showed that both EPP-1 and florfenicol therapy groups harbored substantial *Edwardsiella* in their feeding water and zebrafish feces on day 2 (7.08 and 5.65 log_10_ GC/L of feeding water in FLO and EPP-1 groups, and 8.65 and 7.38 log_10_ GC/g wet feces in the FLO and EPP-1 groups, respectively), but decreased rapidly with feeding time, reaching levels comparable to those in the PBS + SM group (5.14 log_10_ GC/L of feeding water and 4.26 log_10_ GC/g wet feces in the PBS + SM group) (Fig. 2e). We note a rapid decrease of pathogenic *E. piscicida* content in *E. p.* + SM group on day 7, which could be attributed to the effective immunity developed by the surviving zebrafish against the pathogen. On day 4, there was a slight increase in the presence of pathogenic *Edwardsiella* in the *E. p.* + EPP-1 group compared to day 2, suggesting that EPP-1 injected into the fish's body may not always successfully colonized and exert their pathogen-killing efficacy. This could be associated with individual differences in the internal environment of the fish organism. *Edwardsiella* contents in zebrafish feces of the two therapy groups were comparable (4.41 and 4.47 log_10_ GC/g wet feces in FLO and EPP-1 groups, respectively), which were both lower than that in the *E. p.* + SM challenge group on day 7 (6.29 log_10_ GC/g wet feces) (Fig. 2e).

### Phage EPP-1 treatment alleviates oxidative stress and immune responses in the zebrafish gut

**Figure 3:**
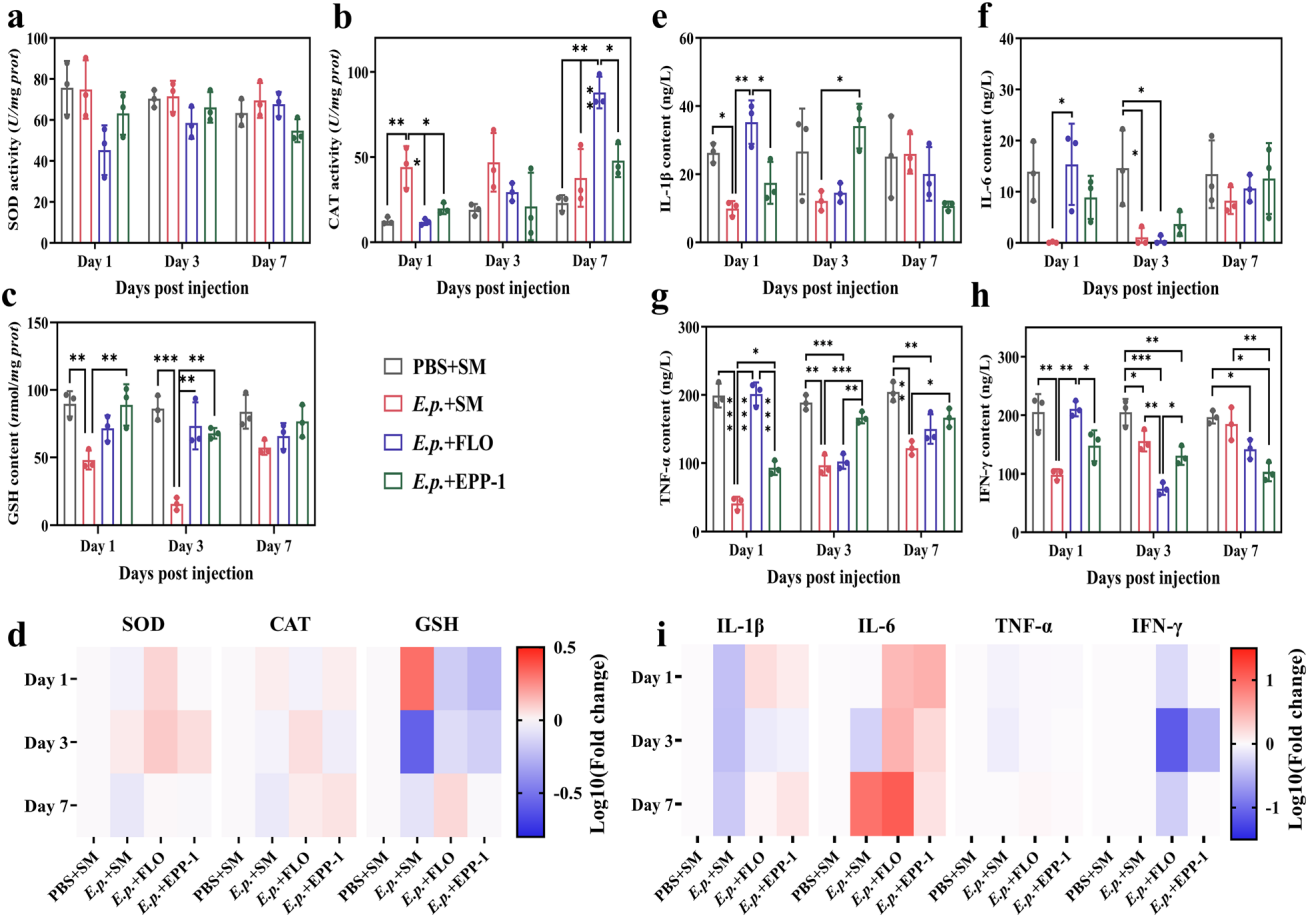
Impacts of different treatments on antioxidant capacity and inflammatory cytokines in the gut and liver of zebrafish. Bar plots of (**a**) SOD activity, (**b**) CAT activity, and (**c**) GSH content in the gut of zebrafish. (**d**) Heat map of SOD activity, CAT activity, and GSH content log_10_ fold changes in the liver of zebrafish. Bar plot of (**e**) IL-1β, (**f**) IL-6, (**g**) TNF-α, and (**h**) IFN-γ content in the gut of zebrafish. (**i**) Heat map of IL-1β, IL-6, TNF-α, and IFN-γ content (log_10_ − transformed) changes in the liver of zebrafish. One-way ANOVA with Sidak’s post-hoc test (a-c, e–h) was performed for significant difference test, *P < 0.05, **P < 0.01, ***P < 0.001. For the heat maps (**d**) and (i), the PBS + SM group served as the baseline for standardization, red representing an up-regulation of enzyme activities or inflammatory cytokines in the gut and liver, and blue representing a down-regulation.

Enzyme activities and inflammatory cytokines in the gut and liver after different treatments were measured to further compare the effects of phage EPP-1 and florfenicol on the antioxidant capacity and immune response in zebrafish (Fig. 3). Compared to florfenicol treatment, phage EPP-1 could efficiently alleviate changes in the CAT activity in the guts of fish infected with *E. piscicida*, especially on day 7 (Fig. 3b). The changes in SOD activity were not significantly altered within 7 days (Fig. 3a). In the liver, there were no significant changes in SOD and CAT activities among the different treatment groups within 7 days; however, the GSH content in the EPP-1 and florfenicol treatment groups decreased significantly on days 1 and 3 compared with that in the negative control group (Fig. 3d).

In addition, we found that phage EPP-1 alleviated immune responses in different organs of zebrafish for a longer time than that in the florfenicol treatment. Specifically, the treatment effect of florfenicol was faster than that of EPP-1 in alleviating the gut immune response caused by *E. piscicida* infection, as reflected by the changes in IL-1β, IL-6, TNF-α, and IFN-γ levels (Fig. 3e-h). In contrast, phage therapy performed better in the middle and later phases of treatment, especially in moderating the response of IL-1β, TNF-α, and IFN-γ on day 3 (Fig. 3e-h). The differences in IL-1β and IL-6 contents in the phage and florfenicol therapy groups became insignificant on day 7 over a prolonged post-injection period (Fig. 3e, f). It was also evident that phage EPP-1 moderated the upregulation of IL-6 levels in the liver compared to the effects of florfenicol (Fig. 3i).

### Composition of bacterial community in the zebrafish gut

**Figure 4:**
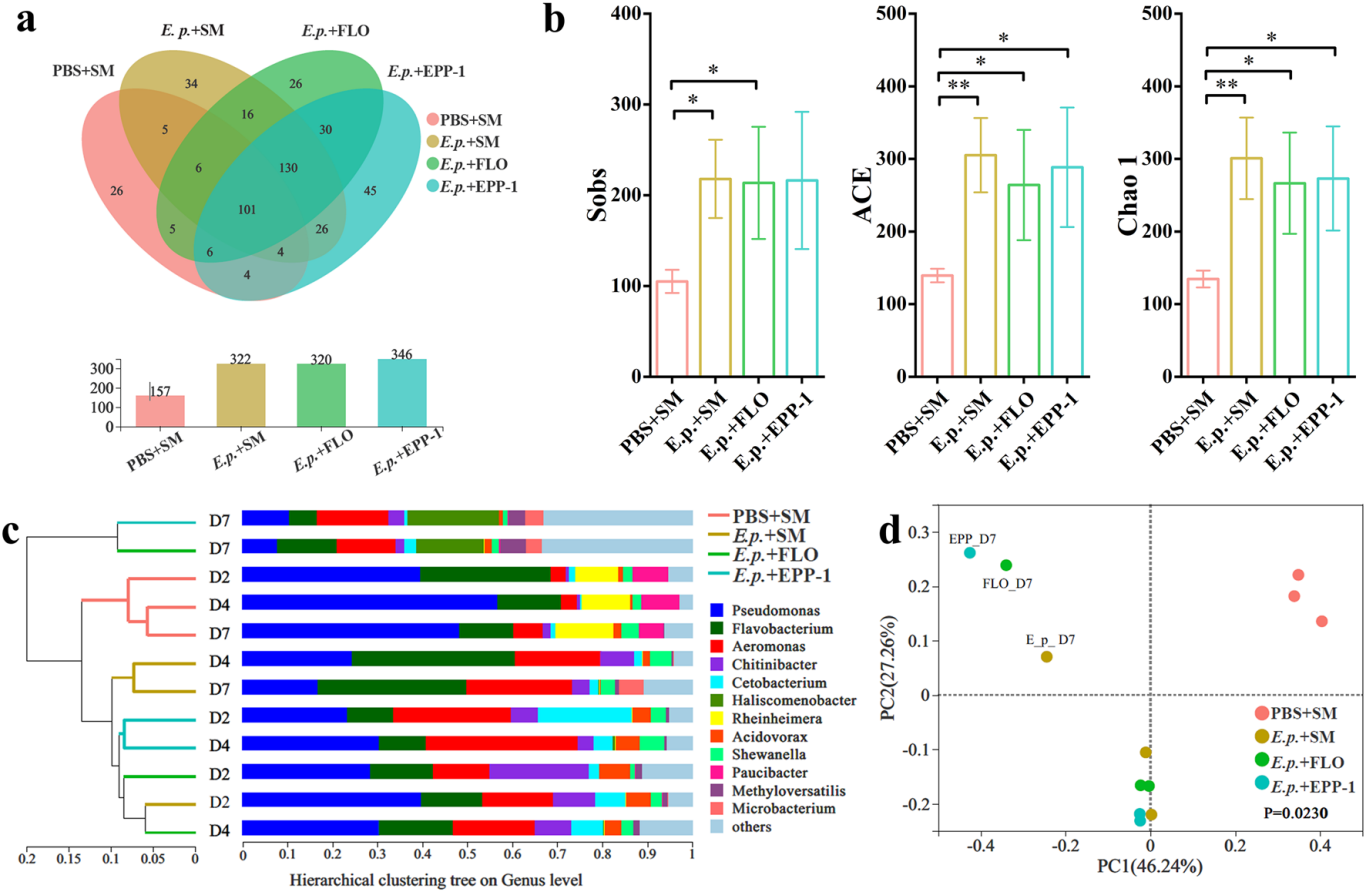
Profiles of the zebrafish gut bacterial community based on 16S rRNA gene under different treatments. (**a**) Venn diagram of OTU numbers in the PBS + SM, *E. p.* + SM, *E. p.* + FLO, and *E. p.* + EPP-1 treatment groups. The bar plot at the bottom shows the total number of OTUs in different groups. (**b**) Comparison of alpha diversity of gut bacterial communities in different treatment groups, including Sobs, ACE, and Chao 1 estimators. *P < 0.05, **P < 0.01, Student’s *t*-test. (**c**) Hierarchical clustering tree based on Bray–Curtis distances at the genus level. The genera with a relative abundance less than 5% are merged into others. (**d**) Principal co-ordinates analysis (PCoA) of the bacterial community of the PBS + SM, *E. p.* + SM, *E. p.* + FLO, and *E. p.* + EPP-1 treatment groups based on Bray–Curtis distances at the OTU level. The analysis of similarities (ANOSIM) was used for statistical testing.

In total, 673,007 optimized sequences were generated through 16S rRNA gene sequencing. The rarefaction curve for each sample tended to become saturated, indicating a sufficient sequencing depth for community composition analysis (Fig. S3a). We identified 464 OTUs, of which 101 were shared by different experimental groups. The number of OTUs in the negative control group was approximately two-fold lower than that in the other groups injected with pathogenic *E. piscicida* (Fig. 4a). Furthermore, the richness (Sobs, Chao 1, and ACE estimators) of the gut bacterial community was significantly lower in the negative control group than in the other experimental groups (Fig. 4b). Proteobacteria and Bacteroidetes were consistently dominant in gut communities, whereas the relative abundance of Fusobacteria in the phage therapy group decreased from 20.89% on day 2 to 0.57% on day 7. Except for the negative control group, all experimental groups had a higher abundance (> 5%) of Actinobacteria on day 7 (Fig. S3b). At the genus level, *Rheinheimera* and *Paucibacter*, the two dominant genera (average relative abundances of 11.08 and 7.25%, respectively) in the negative control communities, almost disappeared in the other groups (Fig. 4c). Compared to that in other gut communities, the relative abundance of *Pseudomonas* declined remarkably in the treatment group communities on day 7, whereas that of *Haliscomenobacter* increased significantly (Fig. 4c).

An analysis of similarity (ANOSIM) showed that different treatments significantly altered the gut bacterial community of zebrafish (*P* = 0.023). According to hierarchical clustering at the genus level, the negative control group without the injection of *E. piscicida* maintained a similar gut bacterial community composition during the entire experimental period. The gut bacterial communities of the phage EPP-1 and florfenicol treatment groups clustered well with those of the positive control group (*E. p.* + SM) at the early stages (days 2 and 4), whereas their gut bacterial communities clustered individually on the day 7, distantly from other communities (Fig. 4c). PCoA and NMDS analyses at the OTU level also revealed a pattern of gut bacterial community composition similar to that of the hierarchical clustering analysis (Fig. 4d and Fig. S3c). Overall, the gut microbiota of the negative control group remained nearly stable and was distinct from that of the other groups. In contrast, the bacterial community in the groups in which pathogens were injected changed dramatically at the end of the experiment (day 7), and interestingly, the phage and antibiotic treatment groups showed a convergent bacterial community shift.

### Functional profiles of the microbial communities of the zebrafish gut

Metagenomic sequencing was used to explore the gut microbial function profiles in different experimental groups, with 6.9 GB of clean data per fecal sample. In total, 1,513,523 non-redundant genes were predicted from 2,779,370 contigs assembled using MEGAHIT. The obtained genes were assigned to 12,142 KEGG orthologies and mainly classified into the following KEGG categories: Global and overview maps, Carbohydrate metabolism, and Amino acid metabolism (Fig. S4a).

**Figure 5:**
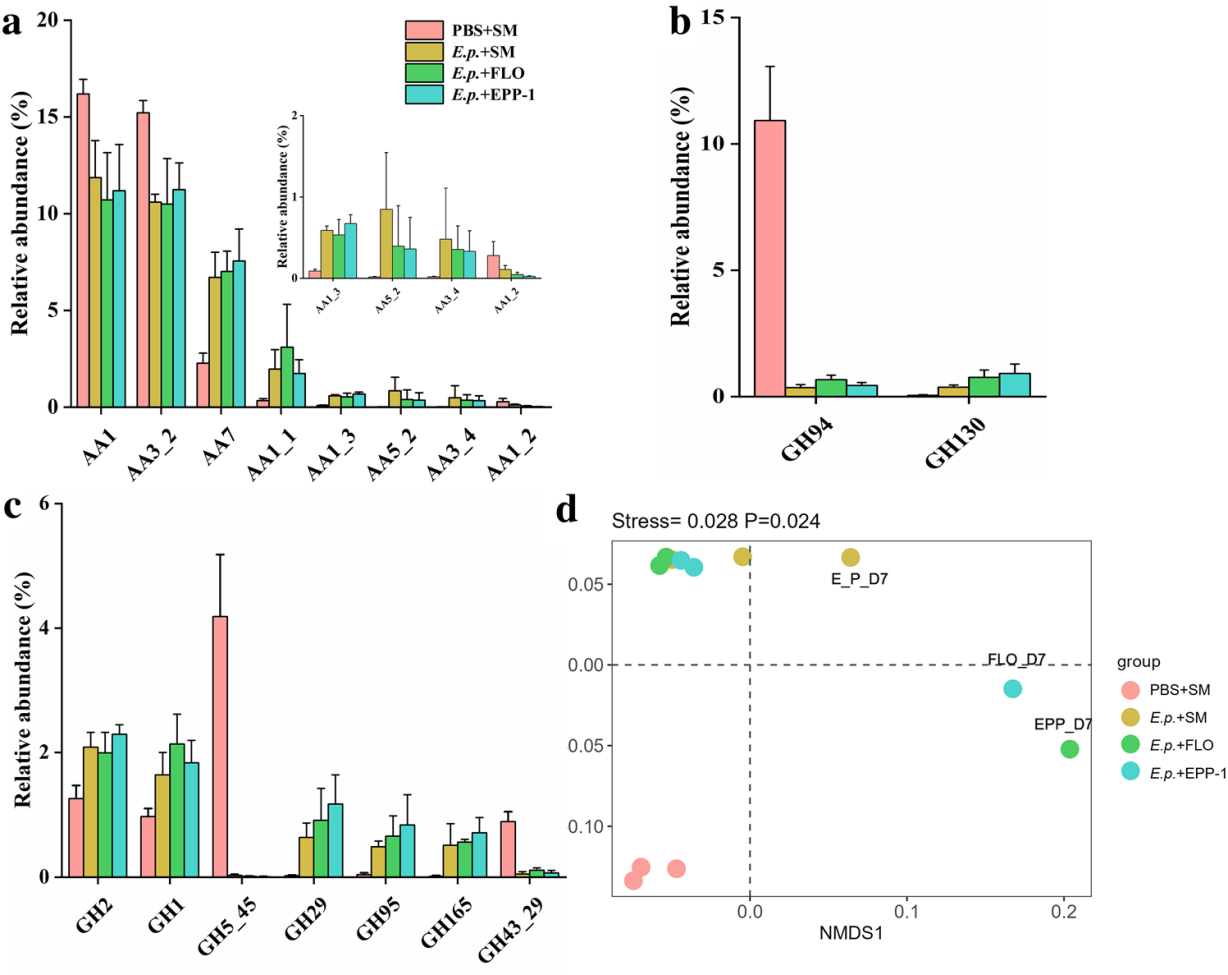
Relative abundance of (**a**) oxidase genes, (**b**) phosphorylase genes, and (**c**) glycosidase genes in four different treatment groups. (**d**) NMDS analysis based on the RPKM values of genes affiliated to CAZyme.

According to the CAZy classification, the affiliated genes (*n* = 496) were mainly distributed among Glycosyl Transferases (GTs), Glycoside Hydrolases (GHs), Carbohydrate Esterases (CEs), and Auxiliary Activities (AAs) families (Fig. S4b). Among them, the RPKM abundances of GH- and AA-related genes were significantly different (*P* = 0.012 and 0.048 for GHs and AAs, respectively) in the four different treatment groups, as revealed through ANOSIM. Specifically, for oxidase genes in the AA family, the abundances of AA7, AA1_1, AA1_3, AA5_2, and AA3_4 in the groups injected with *E. piscicida* were significantly higher than those in the control group, but the abundances of AA1, AA3_2, and AA1_2 were significantly lower (Fig. 5a). Regarding the phosphorylase genes in the GH family, the abundance of GH94 was decreased by 96.73%, 95.90%, and 93.84% in the *E. p.* + SM, *E. p.* + FLO, and *E. p.* + EPP-1 groups, respectively, compared to that in the PBS + SM group (Fig. 5b). In addition, glycosidase abundance in the GH family also changed, with GH5_45 and GH43_29 increasing, whereas the others decreased, in the negative control group (Fig. 5c). Lysozyme GH24 and hydrolase GH153 also increased in the PBS + SM group, but hydrolase GH88 decreased (Fig. S4c). Similar to the pattern of bacterial community dynamics, CAZy-based NMDS analysis revealed that the gut microbial function in the negative control group was different from that in the other groups; whereas the former showed little change, the latter (especially the phage and antibiotic therapy groups) showed large but similar changes on the day 7 (Fig. 5d).

### Phage EPP-1 treatment reduces *floR* gene content

**Figure 6:**
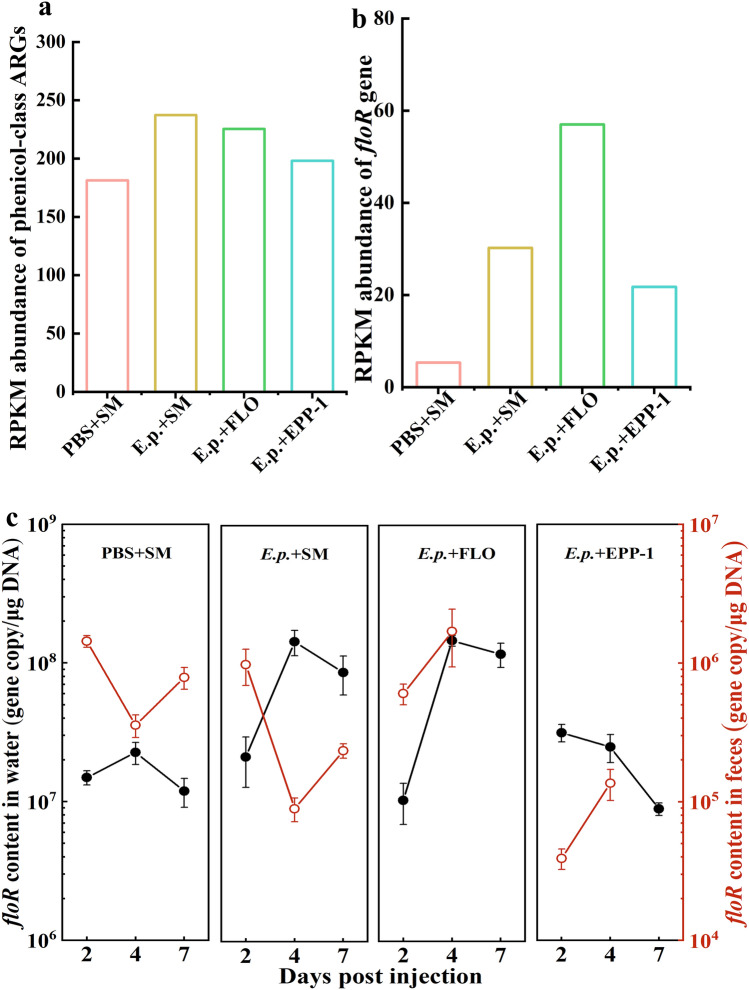
Antibiotic resistance gene (ARG) profiles of different treatment groups based on metagenomic sequencing and qPCR. (**a**) Average RPKM abundance of phenicol-class related ARGs in zebrafish fecal metagenomes from different treatment groups. (**b**) Average RPKM abundance of the *floR* gene in zebrafish fecal metagenomes from different treatment groups. (**c**) qPCR results of *floR* gene in zebrafish feces and feeding water on days 2, 4, and 7. The black line corresponds to *floR* gene content in water and the red one corresponds to *floR* gene content in zebrafish feces.

The most important prospect of phage therapy, as an alternative to antibiotics, is its ability to reduce antibiotic consumption and decrease the risk of antibiotic resistance. In total, 784 ARGs were identified from 12 gut microbial metagenomes, which were dominated by multidrug-, MLS-, glycopeptide-, tetracycline-, peptide-, and beta-lactam-related genes (Fig. S5). The average RPKM abundance of 181.37, 237.40, 225.41, and 198.15 for phenicol-like resistance genes were recognized from the metagenome sequences of the PBS + SM, *E. p.* + SM, *E. p.* + FLO, and *E. p.* + EPP-1 groups, respectively (Fig. 6a). The RPKM abundance of phenicol-class ARGs in the *E. p.* + EPP-1 group was lower than that in the *E. p.* + SM and *E. p.* + FLO groups.

The *floR* resistance gene belongs to the phenicol class and confers bacteria-specific resistance to florfenicol. Bioinformatics analysis identified an average RPKM values of 5.34, 30.22, 57.01, and 21.77 for *floR*-related gene in the metagenomic sequences of the PBS + SM, *E. p.* + SM, *E. p.* + FLO, and *E. p.* + EPP-1 groups, respectively (Fig. 6b). Furthermore, qPCR was used to quantify *floR* gene contents in zebrafish excreta and aquaculture water. Results showed that the *floR* gene content in aquaculture water of the *E. p.* + FLO group increased from 1.04 × 10^7^ GC/μg DNA on day 1 to 1.48 × 10^8^ GC/μg DNA on day 7. In contrast, the gene content in the water of the *E. p.* + EPP-1 group was significantly lower than that of the *E. p.* + FLO group (*P* = 0.0003, two-way ANOVA) and reached a level comparable to that in the PBS + SM group on day 7 (1.19 × 10^7^ GC/μg DNA). In addition, the *floR* gene content in zebrafish feces from the *E. p.* + EPP-1 group was significantly lower than that in the *E. p.* + FLO group (*P* = 0.0127, two-way ANOVA; Fig. 6c). In summary, our results indicate that phage EPP-1 reduces the production of phenicol-class and *floR* ARGs relative to that with florfenicol treatment.

## Discussion

The extensive use of antibiotics induces the residual of antibiotics, the generation of ARGs, and the emergence of ARB, which demonstrate significant health risks. Multiple ARGs and ARB have been detected in aquaculture wastewater and the relevant environments^43,44^. Phages, as antibiotic alternatives, can reduce the antibiotic dosage at source and further alleviate a series of issues caused by antibiotic use. In this study, we have isolated an *Edwardsiella* phage named EPP-1, confirmed its feasibility for edwardsiellosis control in aquaculture, and assessed its impacts on the production of ARGs in relevant habitats.

Relatively few *Edwardsiella* phages have been isolated, the whole genomes of only 14 phages have currently been released at the NCBI. To our knowledge, phage EPP-1 isolated in this study is the first *Edwardsiella* phage carrying the integrase gene and recombinant loci *attR*-*attL* sites required for a lysogenic lifecycle. However, phage EPP-1 demonstrated potent lytic activity against *Edwardsiella piscicida* (MCCC 1K00246). Similarly, phages containing similar lytic characteristics but harboring lysogenic genes have also been isolated from the environment recently^45^. Despite research indicating that temperate phages can be used to treat bacterial infections, for example, Mardiana et al^46^. reported an effective temperate phage for treating *Acinetobacter baumannii* infection in zebrafish, concerns persist regarding the potential for temperate phages to mediate horizontal transfer of pathogenic and antibiotic resistance genes within microbial communities, rendering them generally unsuitable for phage therapy^47^. Interestingly, our results showed that the EPP-1 treatment can effectively reduce the abundance or quantity of the *floR* gene in aquaculture water and feces compared to the florfenicol treatment, suggesting that EPP-1 may not promote the spread of ARGs in intestinal and aquatic microbial communities. Despite this, the application of EPP-1 should be approached with sufficient caution, as it carries lysogeny-related genes, and its potential variability with other strains or under different environmental conditions is not yet fully understood. Recent advancements in sequencing technology and synthetic biology offer new opportunities to explore the use of temperate phages in treating bacterial infections^48,49^. In the future, EPP-1 could be engineered to serve as a safer biocontrol agent by removing the integrase or suppressor from the genome.

In this study, the phage EPP-1 treatment group showed an improved zebrafish survival rate, by 16.7%, relative to that in the challenge group, and this efficacy was comparable to that in the florfenicol therapy group (*P* = 0.2304). Additionally, some studies have suggested that phage therapy can achieve results comparable to those with antibiotics^50^ or other antibiotic alternatives, such as polymyxin B^51^, for fish disease control. Xu and colleagues^52^ obtained an enhanced outcome; a phage cocktail composed of two *Edwardsiella* phages increased the survival rates of zebrafish and turbot by 35%. Interestingly, phage EPP-1 was potent in suppressing host growth even at a low dose (MOI = 0.01) in vitro, but failed to reduce zebrafish mortality in vivo at the same dose. This could be related to complex biological and chemical substances in the zebrafish gut interfering with the phage titer and infection activity. Furthermore, as opposed to florfenicol, phage EPP-1 alleviated the dysregulation of antioxidant capacity and immune system dysfunction in the gut caused by *E. piscicida* infection. Given that phage EPP-1 demonstrates efficacy against *E. piscicida* infection comparable to antibiotics under laboratory conditions, it may hold practical potential as an antibiotic alternative. In future studies, we will further evaluate its therapeutic effectiveness on other economically important fish species, and explore its development as an oral food additive.

The guts of normal animals inherently harbor numerous bacteria and phages in a dynamic balance, and theoretically, the introduction of exogenous phages does not affect the community structure of gut microbes beyond the host due to their strong specificity. Studies have shown that phage administration does not alter the normal intestinal microbiota in humans and mice^53,54^. In aquaculture, the *Aeromonas* phage PZL-Ah152, employed as a biocontrol agent, can reduce the colony numbers of pathogenic hosts, but does not affect the alpha and beta diversity of the crucian carp gut microbiota^55^. In this study, however, we found that survivors from the pathogen injection group (regardless of whether they received antibiotic or phage treatment) exhibited similar composition and function characteristics of gut microbiota, all differing significantly from the control group without *Edwardsiella* infection. The changes in the intestinal microflora might be mainly attributed to the disturbance caused by the injection of the pathogen rather than phage EPP-1. Although the phage treatment effectively inhibits the *Edwardsiella* pathogen (Fig. 2d), similar to the antibiotic florfenicol treatment, neither can restore the gut microbiota structure to its original state in a short period of time.

The dissemination of ARGs arising from the misuse of antibiotics is a major concern to the sustainable development of the aquaculture industry. Our metagenomic and qPCR-based results demonstrated that phage EPP-1 could significantly reduce the levels of *floR* genes in zebrafish excreta and associated environmental waters compared to those with florfenicol. From the perspective of controlling the environmental spread of ARGs, our results indicate that phage therapy for *Edwardsiella* infections in aquaculture is more environmentally friendly compared to antibiotic treatment. In this study, however, phage EPP-1 administered only once by intraperitoneal injection improved survival in zebrafish by only 16.7%. Given the different bacteriolytic activities observed in vitro (Fig. 2a) and in vivo (Fig. 2d), phage viability or titer may be significantly affected by the complex intestinal environment. Furthermore, a single injection regimen may not always maintain sufficient phages within the lesion. And intraperitoneal injection may not be the optimal route of administration, and further comparison with other administration routes such as oral and bath is needed to determine the best mode of administration. The combination of phage with antibiotics or probiotics and the construction of phage cocktails to enhance therapeutic efficacy and reduce the use of antibiotics in a synergistic manner may also be a promising path for phage therapy in aquaculture in the future.

## Conclusions

In this study, we isolated a novel Edwardsiella phage EPP-1 and employed it for the control of *Edwardsiella piscicida* infection in the zebrafish model. Our findings demonstrate that phage EPP-1 achieved therapeutic efficacy comparable to florfenicol, alleviating the dysregulation of antioxidant capacity and immune dysfunction in the zebrafish gut. Importantly, treatment with phage EPP-1 effectively reduced the content of the *floR* resistance gene in zebrafish excreta and aquaculture water. These results suggest that phage therapy holds promise as an effective antibiotic alternative for controlling *E. piscicida* infections in fish, with the potential to mitigate the dissemination of antibiotic resistance genes in aquaculture environments.

### Supplementary Information

Supplementary Information.

## Abbreviations

AA: Auxiliary activity
ANI: Average nucleotide identity
ANOSIM: Analysis of similarity
ARG: Antibiotic resistance gene
CAT: Catalase
CAZy: Carbohydrate-active enZYmes database
CGMCC: China General Microbiological Culture Collection Center
GC: Gene copy
GH: Glycoside hydrolase
GSH: Reduced glutathione
IFN: Interferon
IL: Interleukin
KEGG: Kyoto encyclopedia of genes and genomes
MCCC: Marine Culture Collection of China
NMDS: Non-metric multidimensional scaling
ORF: Open reading frame
OTU: Operational taxonomic unit
PCoA: Principal coordinate analysis
PFU: Plaque forming unit
qPCR: Quantitative polymerase chain reaction
RPKM: Reads per kilobase per million
SOD: Superoxide dismutase
TEM: Transmission electron microscopy
TNF: Tumor necrosis factor
