# Supplementary material for: Bacteriophage EPP-1, a potential antibiotic alternative for controlling edwardsiellosis caused by Edwardsiella piscicida while mitigating drug-resistant gene dissemination
Source: Sci Rep. 2024 Apr 24;14:9399. doi: 10.1038/s41598-024-60214-3 (PMC11043334; doi:10.1038/s41598-024-60214-3)
Supplement: Supplementary file 1 — Supplementary Information. [file 41598_2024_60214_MOESM1_ESM.docx]

# Supplementary Information:

# Bacteriophage EPP-1, a potential antibiotic alternative for controlling edwardsiellosis caused by *Edwardsiella piscicida* while mitigating drug-resistant gene dissemination

Ganghua Han ^a, #^, Ting Huang ^a, #^, Xinchun Liu ^a^, Ruyin Liu ^a, b, *^

^a^ *College of Resources and Environment, University of Chinese Academy of Sciences, Beijing, China.*

^b^ *Yanshan Earth Critical Zone National Research Station, University of Chinese Academy of Sciences, Beijing, China.*

^#^ Ting Huang and Ganghua Han contribute equally to this work as co-first authors.

**^*^** Corresponding author: Ruyin Liu

Address: College of Resources and Environment, University of Chinese Academy of Sciences, No.19(A) Yuquan Road, Shijingshan District, Beijing, P.R. China 100049.

Phone: 86-010-69672961

Email: [Lry1981@ucas.ac.cn](mailto:Lry1981@ucas.ac.cn)

# Supplementary Methods

## Antimicrobial susceptibility test

The overnight culture of *E. piscicida* 1K00246 was diluted to an OD_600_ of 0.4 and further diluted 100-fold. Subsequently, 100 μL of the diluted culture was evenly spread onto TSA plates. After air-drying, the plates were punctured with a sterile puncher. Next, 50 μL of florfenicol solution at concentrations of 80, 40, 20, 15, 10, and 5 μg/mL was added to each well, with an equal volume of ddH_2_O used as the negative control. Additionally, florfenicol sensitivity test paper containing 30 μg florfenicol/piece was placed on the agar plate. The plates were then incubated at 30°C for 24 hours to observe the size of inhibition zones.

## Phage isolation and purification

*Edwardsiella* phage was isolated using the methods reported by Thung^1^ with partial modification. Briefly, the collected environmental water samples were centrifuged at 6000 × g for 10 min to remove debris and coarse particles, the resulting supernatant was then passed through a 0.22 μm pore size filter and subsequently used for phage isolation. The host bacteria *E. piscicida* MCCC 1K00246 in the early exponential phase was incubated overnight with the filtrate and TSB medium at 30℃ and 120 rpm to enrich phages, the mixture was centrifuged and filtered, and then visualize phages by the double layer agar plate method (DLA). The individual plaque was puncture picked up and resuspended in SM buffer, purified 3 to 5 times, and proliferated to 10^9^ PFU/mL for further analysis.

## Transmission electron microscopy

Phage structure and morphology were observed using transmission electron microscopy. Briefly, 3 μL of purified phage particle suspension with titer over 10^9^ PFU/mL was gently placed on carbon-coated 300 mesh copper grids. The grid was then negative stained with 2% (wt/vol) uranyl acetate and air-dried. Phage structure and morphology were visualized with a Transmission electron microscope (JEM-1400, Japan) at an operating voltage of 100 kV. Phage particle dimensions were measured using the ImageJ software^2^.

## Host range test

The traditional spot assay was employed to rapidly and intuitively determine the host range of phage isolated in this study, although this non-EOP (efficiency of plating) method may overestimate the phage host spectrum^3^. Briefly, 3 μL of purified phage suspension (> 10^9^ PFU/mL) was spotted onto lawns formed by different bacterial strains, incubated overnight at 30℃ without shaking, and then observed for plaque formation.

## Temperature and pH tolerance assay

For thermal tolerance assay, equivalent volumes of phage suspension were kept at -80℃, -20℃, 4℃, 25℃, 37℃, 50℃, 65℃, and 80℃ in the refrigerator or water bath for 60 min, respectively, and then the titers were determined by DLA method. Similarly, for pH tolerance assay, 100 μL of phage suspension was added to 900 μL of TSB medium with pH of 1, 3, 5, 7.3, 9, 11 and 13, respectively, and rested for 60 min at 30℃, and then the titer was determined by DLA method. Each experiment was conducted in triplicate.

## Antibacterial effect *in vitro*

The phage storage solution was diluted and mixed with logarithmic growth-phase host *E. piscicida* MCCC 1K00246 and TSB medium at a ratio of 1:1:98 to make their MOI to 100, 10, 1, 0.1, 0.01, and 0, respectively, and incubated at 30℃, 120rpm, and the OD_600_ values were measured every hour with a microplate reader (Synergy^TM^ H1, BioTek Inc., Winooski, VT, USA). MOI of 0 without phages as control and 6 replicates per MOI.

## Phage DNA extraction

Phage nucleic acid extraction was performed as described previously by Kim et al^4^. Briefly, 500 μL of the phage particle suspension was treated with DNase Ⅰ and RNase A (Solarbio, Beijing, China) and incubated at 37℃ for 60 min to degrade the DNA and RNA from *E.* *piscicida* MCCC 1K00246. Ethylenediaminetetraacetic acid (EDTA, Solarbio, Beijing, China) was then added to inactivate the DNase Ⅰ for prevention of the subsequent degradation of phage DNA. Later, proteinase K and 10% SDS solution (Solarbio, Beijing, China) were added, 37℃ water bath for 30 min followed by 56℃ water baths for 60 min, and then a rapid ice bath. DNA purification was performed according to the traditional phenol-chloroform extraction method.

## Bioinformatic analysis

The coding DNA sequences (CDSs) of the phage were predicted and annotated using PHASTER^5^ and the genome profiles of the phage were mapped by CGview Server^6^. The presence of antibiotic resistance genes and virulence factors were screened using the Comprehensive Antibiotic Resistance Database (CARD)^7^ and Virulence Factors Database (VFDB)^8^, respectively. The phylogenetic tree based on terminase large (*TerL*) subunit of phage was constructed using the neighbour-joining method in MEGA X with 1000 bootstrap replications^9^. To avoid bias from single gene, whole genome-wide average nucleotide identity (ANI) values were also calculated through the online platform JspeciesWS (<http://jspecies.ribohost.com/jspeciesws/>) and visualized on GraphPad Prism 9.

## Determination of pathogenic infection dose and phage therapeutic dose

To assess the pathogenicity of *E.* *piscicida* MCCC 1K00246 and the efficacy of phage EPP-1, it is warranted to determine the pathogen infection dose and the phage therapeutic dose. Four groups of different infectious doses were set up with 10 zebrafish per tank and zebrafish in each tank were injected intraperitoneally with 2 μL of different CFU of bacterial solution (dilution with sterile PBS buffer) making the injection dose of each group to 0, 10^4^, 10^5^, and 10^6^ CFU/fish, respectively. In which, the 0 CFU/fish group was injected with 2 μL sterile PBS buffer and served as the control group. The mortality rate was recorded daily for 15 consecutive days. To highlight the efficacy of phage therapy, a dose with mortality of no less than 50% was selected for subsequent experiments.

Therapeutic phages were injected intraperitoneally simultaneously with a dose of bacterial infection identified, making their MOI to 0, 0.1, 1, 5, and 10, respectively. High titer phage lysate was diluted with SM buffer. In which, the MOI=0 group was injected with 1 μL sterile PBS buffer and 1 μL sterile SM buffer. The mortality rate was recorded daily for 10 consecutive days and the MOI with optimal therapeutic effect was selected for the further trials. The breeding conditions are standard aquaculture system, as shown in the text.

## Quantitative PCR

The *gyrB* and *floR* genes were quantified in water and stool samples in triplicate against a 10-fold serial dilution standard curve and a DNA-free negative control. Results of the assays were analyzed based on the standard curve of each gene. Briefly, amplification of *gyrB* gene was performed in a 20 μL reaction volume containing 10 μL of 2 × SYBR® Premix Taq^TM^ (TaKaRa, Japan), 0.5 μL of each primer (10 μmol/L), 1 μL of template DNA, 0.4 μL of bovine serum albumin (5 mg/mL, Solarbio, Beijing, China), and 7.6 μL of ddH_2_O using an ABI QuantStudio™ 1 real-time PCR instrument (Applied Biosystems, Grand Island, NY, USA). The thermal cycle consists of 30 s initial denaturation at 95℃, followed by 40 cycles of the following: 10 s at 95℃, 20 s at 63℃, and 20 s at 72℃, with reading plate after each cycle. The setting of the amplification melting curve to verify the absence of non-specific amplicons are as follows: 15 s at 95℃, 15 s at 60℃, and 15 s at 95℃. For the amplification of *floR* gene, a 20 μL reaction system containing 10 μL of 2 × SYBR® Premix Taq^TM^ (TaKaRa, Japan), 0.2 μL of each primer (10 μmol/L), 1 μL of template DNA, 0.4 μL of bovine serum albumin (5 mg/mL, Solarbio, Beijing, China), and add ddH_2_O to the required volume. The thermal cycle for this amplification consists of 10 min initial denaturation at 95℃, followed by 40 cycles of denaturation at 95℃ for 15 s, annealing at 56℃ for 15 s, and extension at 72℃ for 1 min, and the melting curve was performed under default settings.

## PCR for 16S rRNA gene

The amplification of V3–V4 hypervariable regions of bacteria was performed in a 20 μL reaction volume containing 4 μL of 5 × FastPfu Buffer, 2 μL of 2.5 mM dNTPs, 0.8 μL of each primer (5 μM), 0.4 μL of FastPfu Polymerase, 0.2 μL of BSA, 10 ng of template DNA, and add ddH_2_O to 20 μL using an ABI GeneAmp® 9700 instrument (Applied Biosystems, Grand Island, NY, USA). The thermal cycle consists of 95℃ for 3 min, followed by 29 cycles at 95℃ for 30 s, 53℃ for 30 s, and 72℃ for 45 s and extension at 72℃ for 10 min. The PCR products were identified by 2% agarose gel electrophoresis and further perform amplicon sequencing.

# Supplementary Tables

Table S1. Bacterial strains and host range of EPP-1

| Bacterial species | Strain ID | Isolation source | *floR** | Lysis effect^#^ |
| --- | --- | --- | --- | --- |
| *Edwardsiella anguillarum* | DSMZ 27202 | Diseased Japanese eel | - | - |
| *Edwardsiella hoshinae* | ATCC 33379 | Dead female puffin | - | - |
| *Edwardsiella ictaluri* | ATCC 33202 | Channel catfish | - | - |
| *Edwardsiella ictaluri* | MCCC 1K00244 | Striped catfish | - | - |
| *Edwardsiella piscicida* | MCCC 1K00245 | Lantern fish | - | - |
| *Edwardsiella piscicida* | MCCC 1K00246 | Turbot | - | host |
| *Edwardsiella piscicida* | MCCC 1K03230 | Diseased sturgeon | - | + |
| *Edwardsiella tarda* | ATCC 15947 | Feces | - | - |
| *Edwardsiella tarda* | MCCC 1K00241 | Catfish | - | + |
| *Edwardsiella tarda* | MCCC 1K00243 | Marble cichlid | - | - |
| *Edwardsiella sp.* | MCCC 1K00239 | Marbled eel | - | + |
| *Edwardsiella sp.* | MCCC 1K00240 | American eel | - | + |
| *Edwardsiella sp.* | MCCC 1K00242 | Marbled eel | - | + |

All bacterial strains used in study were obtained from the MCCC.

* PCR detection of the *floR* gene. (-): no; (+): yes.

^#^ Presence of plaque. (-): no; (+) yes.

Table S2. Water quality and measurement methods of feeding water

| Parameters | Methods^*^ | Water quality |
| --- | --- | --- |
| Temperature (℃) | HACH multiparameter | 23.45±0.55 |
| pH | HACH multiparameter | 8.31±0.05 |
| ORP (mV) | HACH multiparameter | -89.11±3.58 |
| Hardness (mg CaCO_3_/L) | Colorimetric with test strips | 250–375 |
| DO (mg/L) | HACH multiparameter | 8.42±0.16 |
| TDS (μS/cm) | TDS meter | 593.25±30.34 |
| NH_4_^+^-N (mg/L) | HACH Lange cuvette kit | 0.01±0.02 |

*HACH multiparameter: HACH DR1900, USA; Colorimetric with test strips: Total Hardness Test, Millipore, Bedford, MA, USA; TDS meter; Sartorius, Germany

Table S3. Primers and corresponding profiles for PCR or qPCR

| Gene | Primer | Sequence (5’–3’) | Product size |
| --- | --- | --- | --- |
| 16S V3–V4 | 338F | ACTCCTACGGGAGGCAGCAG | 468bp |
|  | 806R | GGACTACHVGGGTWTCTAAT |  |
| *gyrB* | F | TGGCGACACCGAGCAGA | 207bp |
|  | R | ACAAACGCCTTAATCCCACC |  |
| *floR* | F | CGGTCGGTATTGTCTTCACG | 171bp |
|  | R | TCACGGGCCACGCTGTAT |  |

# Supplementary Figures


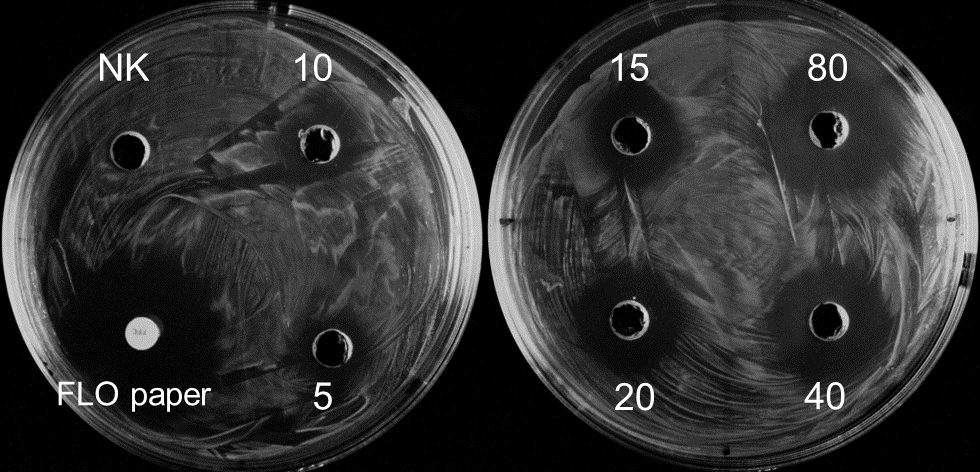


Fig. S1. Antimicrobial susceptibility test of E. piscicida MCCC 1K00246 to different concentrations of florfenicol. Florfenicol concentration: μg/mL; FLO test paper: 30 μg florfenicol/piece; NK: no florfenicol added.


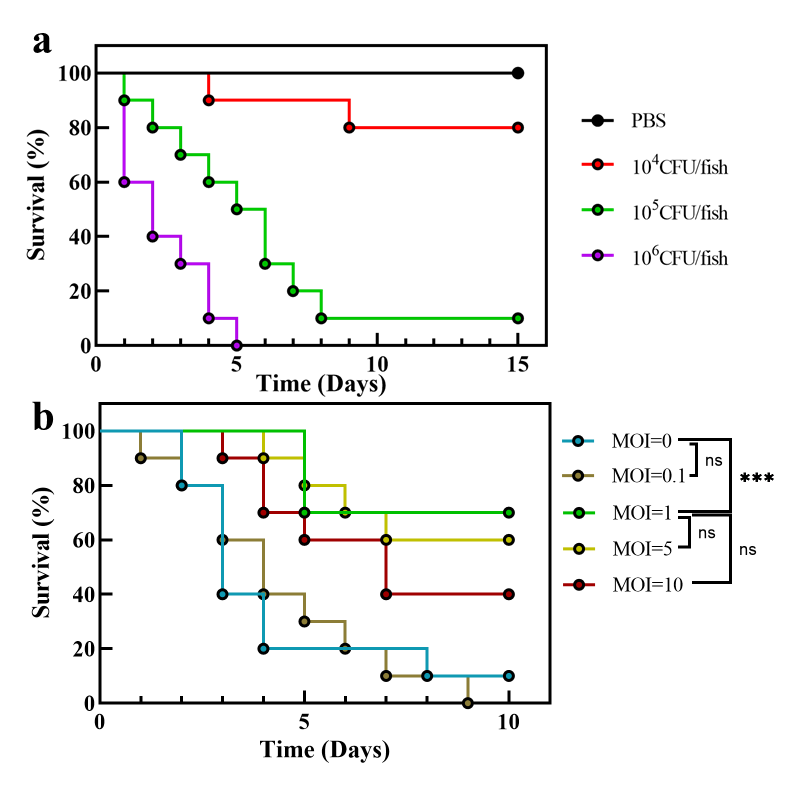


Fig. S2. (a) Survival curves of zebrafish under different infection doses within 15 days (n=10). (b) Survival curves of zebrafish under different MOIs at the infection dose of 10^5^ CFU/fish within 10 days (n=10, **P*<0.05, ***P*<0.01, ****P*<0.001, *****P*<0.0001, Mantel-Cox test). The mortality of zebrafish at the infection dose of 10^5^ CFU/fish was 90% within 15 days, which is an ideal dose for follow-up studies compared to the infected dose of 10^4^ or 10^6^ CFU/fish that result in either excessively low or high mortalities, and selected for further studies. The phage treatment dose of MOI=1 was the most effective of the four doses selected, and therefore, it was chosen for efficacy comparison with florfenicol.


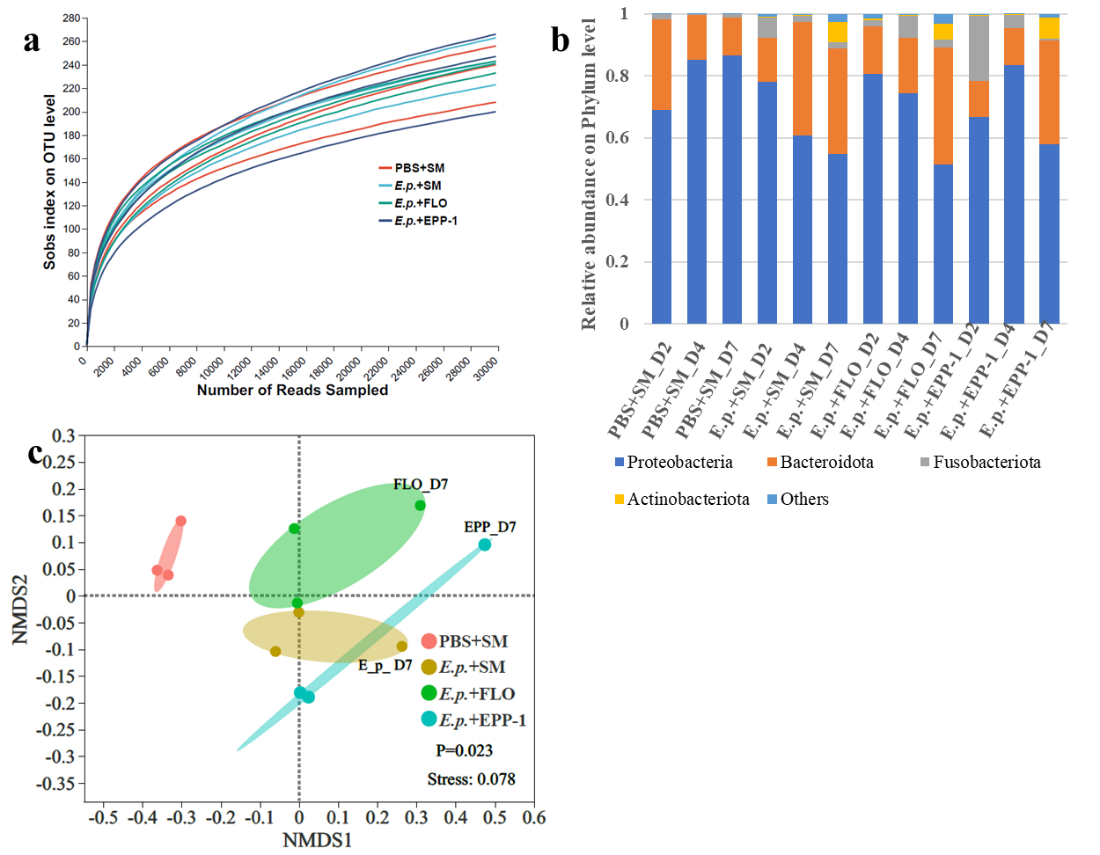


Fig. S3. (a) The tending saturation rarefaction curves for each sample indicating their sufficient sequencing data for further analysis. (b) Relative abundance of bacterial composition of each sample on Phylum level. Only 4 major Phylum are listed in the figure, and the rest are merged into others. (c) NMDS analysis of the bacterial community of the PBS + SM, *E. p.* + SM, *E. p.* + FLO, and *E. p.* +EPP-1 groups on OTU level based on Bray-Curtis distance. The ANOSIM test was used for statistical test.


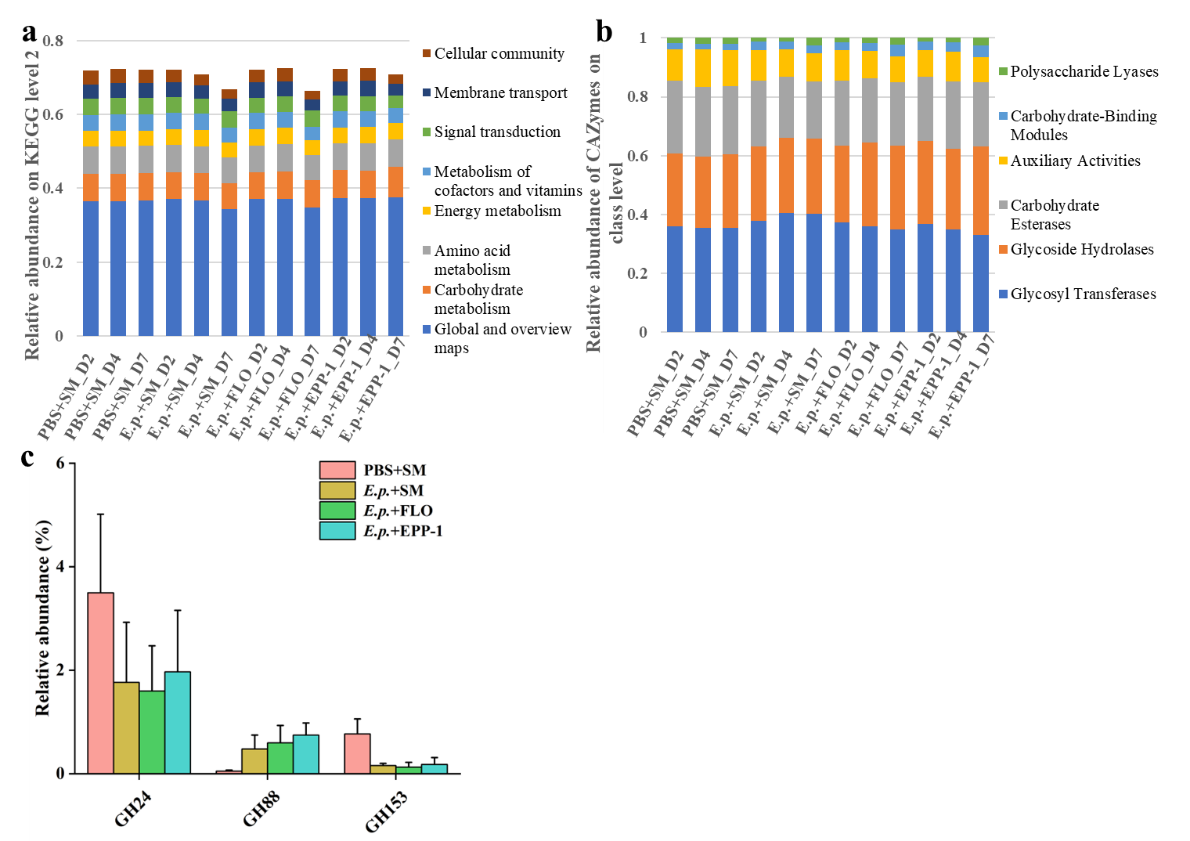


Fig. S4. (a) Relative abundance of gut microbial function of each sample on KEGG level 2. Only 8 major functions are listed in the figure. (b) Relative abundance of top 6 carbohydrate active enzymes of each sample on class level. (c) Relative abundance of lysozyme GH24 and hydrolase GH153, and hydrolase GH88 in four different treatment groups.


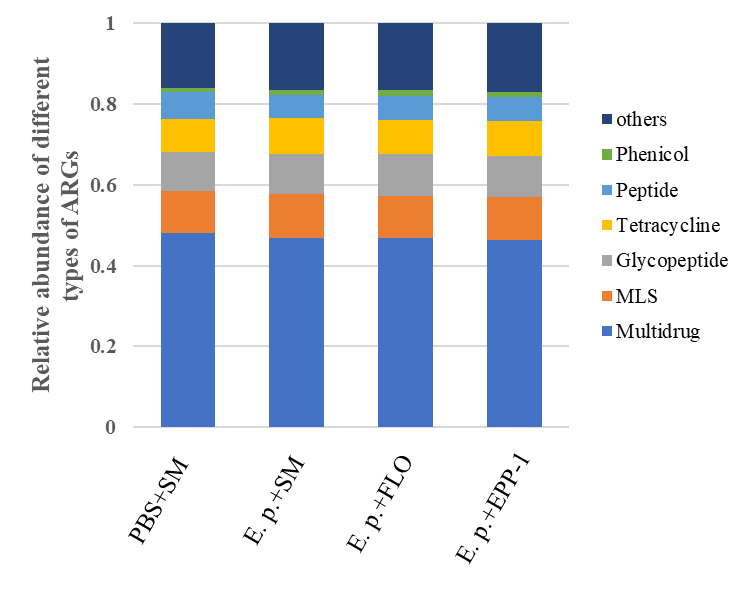


Fig. S5. Relative abundance of different types of ARGs detected in each sample. The types with relative abundance lower than 5% are merged into others, but the ARGs of the Phenicol class are shown in the figure.

# Reference:

1 Thung, T. Y. *et al.* Isolation of food-borne pathogen bacteriophages from retail food and environmental sewage. *Int. Food Res. J.* **24**, 450-454 (2017).

2 Schneider, C. A., Rasband, W. S. & Eliceiri, K. W. NIH Image to ImageJ: 25 years of image analysis. *Nat. Methods* **9**, 671-675 (2012).

3 Khan, M. M. & Nilsson, A. S. Isolation of phages for phage therapy: a comparison of spot tests and efficiency of plating analyses for determination of host range and efficacy. *PLoS One* **11**,10(3):e0118557 (2015).

4 Kim, S. G. *et al.* Isolation and characterisation of pVa-21, a giant bacteriophage with anti-biofilm potential against *Vibrio alginolyticus*. *Sci. Rep.* **9**, 6284 (2019).

5 Arndt, D. *et al.* PHASTER: a better, faster version of the PHAST phage search tool. *Nucleic Acids Res.* **44**, W16-W21 (2016).

6 Grant, J. R. & Stothard, P. The CGView Server: a comparative genomics tool for circular genomes. *Nucleic Acids Res.* **36**, W181-W184 (2008).

7 Alcock, B. P. *et al.* CARD 2023: expanded curation, support for machine learning, and resistome prediction at the Comprehensive Antibiotic Resistance Database. *Nucleic Acids Res.* **51**, D690-D699 (2023).

8 Liu, B., Zheng, D. D., Jin, Q., Chen, L. H. & Yang, J. VFDB 2019: a comparative pathogenomic platform with an interactive web interface. *Nucleic Acids Res.* **47**, D687-D692 (2019).

9 Kumar, S., Stecher, G., Li, M., Knyaz, C. & Tamura, K. MEGA X: Molecular Evolutionary Genetics Analysis across Computing Platforms. *Mol. Biol. Evol.* **35**, 1547-1549 (2018).
